# A plesiotherapy technique for the post‐operative treatment of skin cancer using Ir192 microSelectron.

**DOI:** 10.1120/jacmp.v9i4.2890

**Published:** 2008-10-29

**Authors:** Ioannis Abatzoglou, Pelagia Tsoutsou, Michael I. Koukourakis

**Affiliations:** ^1^ Department of Radiotherapy and Oncology Democritus University of Thrace Alexandroupolis Greece

**Keywords:** plesiotherapy, skin cancer, radiotherapy, high dose rate, Ir192

## Abstract

We describe a technique of postoperative irradiation of skin cancer using plesiotherapy with Ir192 high dose rate microSelectron afterloading system (Nucletron, Veenendaal, Netherlands). The clinically defined area is drawn on the skin and the flexible ‘skin applicator’ is then orientated so that the drawn skin area is encompassed within the catheter defined surface. Using a thin pewter wire, the skin drawn area is copied on the air‐adjacent surface of the applicator. *Ex vivo* CT simulation follows. The data are then transferred to the radiotherapy planning computer and the catheters are virtually reconstructed. The isodose curve chosen to prescribe the dose is 3 mm to 5 mm away from the skin surface. Three fractions of 8Gy are scheduled, 1 week apart, delivering a radiobiological equivalent of 48Gy of standard radiotherapy within 2 weeks. Our preliminary experience shows excellent early skin tolerance. The study is ongoing to assess efficacy and late effects.

PACS number: 87.53.Jw

## I. INTRODUCTION

Skin cancer (basal cell and squamous cell carcinomas) is a common human neoplasia strongly linked to the chronic exposure to oxidative stress and solar radiation.^(1)^ Surgical excision provides excellent local control,^(1)^ ^,^ ^(2)^ ^,^ ^(3)^ while radiotherapy is an effective alternative providing high cure rates depending upon local stage.^(4)^ Ipsilateral neck dissection and/or radiotherapy of the regional nodes is recommended in cases of clinically involved nodes or in cases with large tumors as metastasis rate increases with tumor size.^(5)^

External beam radiotherapy with kilo‐voltage irradiation (40–180kV) or electron beams (2–18MeV) provide excellent control rates, while interstitial radiotherapy with needle implantation has also been used in specialized centers with similarly good results.^(6)^ Postoperative radiotherapy at the site of local excision is also recommended to eliminate residual cancer cells after subtotal resection, or whenever the pathology report indicates infiltrated surgical margins.

Here we report a technique we use in our department for postoperative irradiation after incomplete surgical removal of a skin carcinoma using plesiotherapy applied with a special applicator designed for the Ir192 microSelectron high dose rate brachytherapy unit.

## II. MATERIALS AND METHODS

### A. Preparation for planning

**Figure 1:**
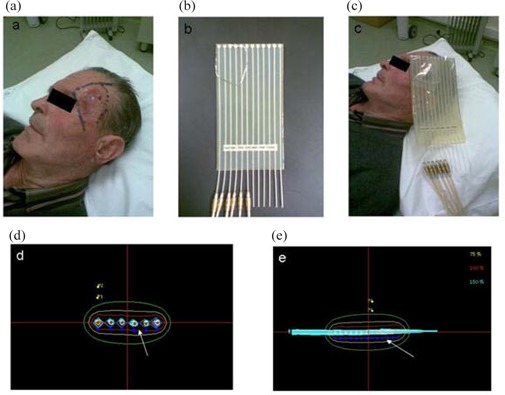
A technique of postoperative plesiotherapy with high dose rate Ir192 for skin cancer: (the area to be irradiated is drawn on the skin of the patient; (1b) the ‘skin applicator’; (1c) the applicator is fixed on the skin at the prescribed area; (1d) transverse and (1e) longitudinal sections of the applicator and the isodose curves around the sources. The red curve (indicated by an arrow) is the 100% isodose curve calculated 3 mm from the skin surface of the applicator.

The patient's surgically treated skin area is thoroughly examined and palpated to clinically define the margins and shape of the area to irradiate. This area is carefully drawn on the skin Fig. 1(a). Specific areas considered as high risk areas to host residual tumor can be additionally drawn within the initially circumscribed area.

The flexible ‘skin applicator’ designed for the high dose rate Ir192 microSelectron (Nucletron, Veenendaal, Netherlands) is then placed on the skin. This applicator has a rectangular shape Fig. 1(b) and is transparent, with a thickness of 8 mm. It hosts 12 catheters of 2 mm in diameter located at the central level (4 mm from the skin surface and 4 mm from the air‐adjacent surface). The distance between catheters is 1 cm. The applicator is orientated to a desired direction so that: (i) it can be well fixed on the skin, and (ii) the drawn skin area is encompassed within the catheter defined surface Fig. 1(c). The flexibility of the applicator allows the catheters to follow the curvatures of the body surface. Using a thin pewter wire, the skin drawn area is copied on the air‐adjacent surface of the applicator Fig. 1(c). The patient's skin is marked appropriately so that the applicator can be removed and reliably repositioned.

### B. Radiotherapy planning

The applicator is removed and scanned using a CT simulator, obtaining 5 mm thick slices (*ex vivo* simulation). In this way there is a minimal discomfort for the patient. However, if necessary, the patient with the mounted applicator can be scanned so the critical organs (e.g. eye lens) can be recognized and the dose to them can be calculated. No wire mold is inserted in the plastic catheters, since these are well recognized in the CT images as small round air spaces.

The data are then transferred to the PLATO radiotherapy planning computer (Nucletron, Veenendaal, Netherlands). The catheters are virtually reconstructed and then virtually loaded at the points corresponding to the skin area we prescribed for irradiation. In‐field areas can be also recognized and a second plan can be performed to deliver a booster radiation dose to such high recurrence risk areas. The treatment planning is performed and isodose distribution is displayed in 3D. The dose is prescribed at 3 mm to 5 mm from the skin surface for postoperative radiotherapy of the head, where bone structures are immediately under the thin skin. For other locations, depending upon the surgical scar thickness and the absence of bone structures immediately under the skin, the dose can be prescribed to points up to 1 cm from the skin surface. The dwell times are optimized to a surface at the prescribed distance from the skin surface. When residual or recurrent tumor is evident, the isodose distribution can be shaped to allow deeper distribution of the 100% curve within the area of the tumor, keeping a shallower distribution in the macroscopically intact areas. The radiotherapy plan is automatically transferred to the microSelctron's control computer.

### C. Dose prescription

The isodose curve chosen to prescribe the dose is 3 mm to 5 mm depth in tissue from the skin surface area Fig. 1(d), 1(e). Three fractions of 8Gy are scheduled, 1 week apart. The dose rate at the 100% isodose curve in this particular plan is 2.97cGy/s. The total dose of 24Gy (3×8 Gy) has a radiobiological equivalent of 48Gy of standard radiotherapy (for tissue α/β ratio of 4Gy) delivered within 2 weeks.^(7)^ If necessary, specific regions within the treated area can receive a booster dose of 2Gy so that higher biological dose is reached.

### D. Treatment

The patient is escorted to the treatment room and placed in a sitting or lying position as necessary to ensure a comfortable stand of the head or body to avoid undesirable movement of the irradiated area. The applicator is then placed and fixed at the predesigned skin area Fig. 1(c). The applicators are connected to the microSelectron system and therapy is initiated. The microSelectron uses a flexible wire to forward the iridium source in the applicators. Treatment lasts 3 min to 10 min depending upon the field size and the source activity. The applicator is removed and the patient is free to return home.

Our preliminary experience shows excellent early skin tolerance. The study is ongoing to assess efficacy and late effects.
